# Neutral public good mechanisms

**DOI:** 10.1371/journal.pone.0266278

**Published:** 2022-04-04

**Authors:** Jin Yeub Kim

**Affiliations:** School of Economics, Yonsei University, Seoul, Republic of Korea; Peking University, CHINA

## Abstract

In this paper, I justify neutral mechanisms as the reasonable solutions for public good provision and cost shares in public goods problems. I illustrate that neutral mechanisms can be easily computed by the tractable set of conditions with straightforward interpretations for a class of public goods problems. I show that, unlike neutral mechanisms, ex ante incentive efficient mechanisms are not robust to a perturbation of the information structure at the time of mechanism selection. I highlight several merits of using neutral mechanisms instead of interim incentive efficient mechanisms: Neutral mechanisms yield sharp predictions, are invulnerable to the possibility of information leakage during the selection process, and have the attractive properties of both efficiency and equity. I discuss implications for the analysis of ex ante and interim incentive efficient mechanisms for public goods problems.

## Introduction

In the classic public goods problem, agents must decide whether or not to produce a public good and how to divide the cost of production. This paper considers environments where each agent’s valuation of the public good is private information or type, and the agents themselves may agree on a decision rule or mechanism to help them make decisions. Which mechanisms should we expect to be selected and used by the agents? That is, what would be considered a reasonable set of predictions for mechanism selection that has a strong predictive power as well as attractive properties?

By the revelation principle, attention should be restricted to mechanisms that are feasible in the sense that the agents are willing to participate and to reveal their private types honestly in the mechanism. Given the set of feasible mechanisms, the concept of Pareto efficiency is clearly a minimal requirement for defining reasonable selections by the agents. Two notions of efficiency are relevant and can be applied to incomplete information settings: ex ante incentive efficiency and interim incentive efficiency [1].

A mechanism is *ex ante incentive efficient* if it is feasible and there is no other feasible mechanism that is preferred by all agents in terms of their ex ante expected payoffs (i.e., what they expect before they learn their private information). A mechanism is *interim incentive efficient* if it is feasible and there is no other feasible mechanism that is preferred by all types of all agents in terms of their interim expected payoffs (i.e., what they expect when each agent knows his private type but not any other agent’s type). (For exposition, I use male pronouns for an agent.) The goal of this paper is to show that delimiting the ex ante or interim incentive efficient mechanism as the “solution” is problematic and instead characterizing neutral mechanisms [2] (as will be defined) for public good provision is more theoretically appealing and reasonable from efficiency and equity standpoints.

My formal analysis begins by setting up a two-person Bayesian bargaining problem to represent public good environments with incomplete information. I restrict attention to two players; this substantially simplifies the exposition while conveying all the main insights. In this problem, two players can jointly choose among possible decisions about whether a discrete public good should be produced, and if so, how much each player should pay for producing it. A discrete public good problem concerns a 0-1 public good decision, i.e., a public good is either produced in full or not produced at all. A player’s type is a complete description of his private information about his valuation for the public good, and each player has beliefs over the other player’s possible types. A mechanism for this problem chooses the decision as a function of the players’ independently and confidentially reported types. I assume that the players are able to negotiate with each other for which mechanism to implement among all feasible mechanisms.

Given that attaining efficiency is a minimal requirement, the efficient solution for the public goods problem can be contrasted in two cases: (i) the players choose a mechanism before learning their private information (ex ante stage) and (ii) the players choose a mechanism after they learn their own private information (interim stage). If the players can choose a mechanism at the ex ante stage, then they would agree on a mechanism that is ex ante incentive efficient. Selecting an ex ante incentive efficient mechanism is appropriate for maximizing the probability of the public good being produced in situations where it appears to be worth more than it costs. If the mechanism is selected at the interim stage, then a chosen mechanism would be interim incentive efficient. Because ex ante incentive efficiency implies interim incentive efficiency [1], any selection among the set of interim incentive efficient mechanisms may be deemed reasonable when the only concern is achieving efficiency for public good provision.

I show that the set of ex ante incentive efficient mechanisms is not robust to a perturbation of the ex ante informational structure at the time of mechanism selection. There are other issues with selecting an ex ante incentive efficient mechanism as the solution for optimal provision of public good. For example, the selection of such mechanism may not be implementable if the players can renegotiate their mechanism once they learn their private information. Also, it is often the case that the players know their own valuations for the public good before they negotiate. (I thank an anonymous reviewer for pointing out the latter issue.) My paper focuses on a more conceptual issue with the information structure at the time of selection.

To show that the set of ex ante incentive efficient mechanisms is not robust, I consider a perturbed setting where the players are not absolutely certain that nobody has any private information at the time when they meet initially to select a mechanism. I call this stage of mechanism selection *almost ex ante*, which indicates informational environments in between the ex ante and interim ones. (I thank Roger Myerson for suggesting this term. The probability of being informed can be any value between zero and one, so the almost ex ante stage can be alternatively called an almost interim stage.) When the selection is made at the almost ex ante stage, the set of incentive efficient mechanisms should consist of those feasible mechanisms that incorporate efficient aggregation of both the interim preferences of the players who have private information and the ex ante preferences of those who do not. I find that this set coincides with the set of interim incentive efficient mechanisms and thus is a superset of the set of ex ante incentive efficient mechanisms. Therefore, the focus on an ex ante incentive efficient mechanism as the most reasonable mechanism for players to choose is valid only when there is absolutely no doubt that all players do not know their types at the selection stage. If any doubt exists, then reasonable selections must be defined on a larger set of interim incentive efficient mechanisms.

I then discuss two possible cautions in focusing on the set of interim incentive efficient mechanisms as a reasonable set of predictions. First, the set of interim incentive efficient mechanisms is typically quite large, so characterizing this set generates indefinite predictions of public good mechanisms that may arise. Second, selecting a particular interim incentive efficient mechanism is vulnerable to the possibility of information leakage, which will be later discussed in detail. Therefore, the set of interim incentive efficient mechanisms is too weak to be the solution set for the public goods problem.

These results call for some other solution criterion to delimit reasonable predictions of mechanisms that are sufficiently sharp, robust to alternative specifications of the information structures at the time of mechanism selection, and invulnerable to the issue of information leakage from the selection of the mechanism. The concept of neutral mechanism, proposed by Myerson [2], not only satisfies those desiderata but also has the desirable properties of both efficiency and equity. The neutral mechanism is defined as the minimal set of feasible mechanisms that satisfies probability invariance, extension, and random dictatorship axioms, which I do not scrutinize here. Myerson [2] showed that a neutral mechanism can be characterized as a feasible mechanism that is efficient and equitable in terms of players’ virtual utilities that incorporate what they would have wanted if they were of different types. In particular, the neutral mechanism equalizes virtual utilities of the players, where the virtual utilities are derived from a maximization problem to maximize the weighted sum of the players’ expected utilities.

In public goods problems, the decision to produce a public good cannot be separated from the decision on dividing the cost of production; so in many situations, attaining both efficiency and fairness of production and cost sharing is concerned and important. The concept of neutral mechanism selects an equitable mechanism on the efficient frontier of the set of feasible mechanisms, so it can be considered a reasonable requirement for the solution to the problem of public good provision. I illustrate how neutral mechanisms can be easily characterized with straightforward interpretations in a class of public goods problems. I also discuss why the application of neutral mechanisms is a theoretically and intuitively more appealing way than ex ante or interim incentive efficiency to represent reasonable choices of public good mechanisms that may actually arise in practice.

### Related literature

This paper connects with three lines of research. First, the most closely related literature is a series of papers by Ledyard and Palfrey [3–6]. Ledyard and Palfrey fully characterized interim incentive efficient mechanisms for the provision of public goods in Bayesian environments [3, 4]; compared the performance of simple voting rules with that of interim incentive efficient public good mechanisms [5]; and provided a more general framework to study the properties of interim efficient mechanisms for the class of linear independent environments [6]. See also Gresik [7] and Wilson [8] who explored interim incentive mechanisms for sealed-bid trading problems.

In terms of the model and applications, my paper shares several common features with Ledyard and Palfrey’s papers. Ledyard and Palfrey [3] considered a simple case with two types and a 0-1 public good decision; likewise, I work with finite type sets and a 0-1 public good decision. In the models of [4, 5] with a continuum of types, the individuals decide on a level of a public good; but with the linear production technology, the optimal level of the public good will always be either 0 or 1, so this is essentially equivalent to making a 0-1 public good decision. The key difference is that my analysis makes use of the concept of neutral mechanism, developed by Myerson [2], which is a refinement of interim incentive efficiency. Given that the literature on the neutral mechanism is slim and that there are only few applications using the concept (for example, see Balkenborg [9], Balkenborg and Makris [10], De Clippel and Minelli [11], and Kim [12, 13]), I regard its application to a Bayesian public good environment as a major contribution of this paper.

In a broader sense, this paper relates to the vast literature on bargaining solution concepts and mechanism design problems for Bayesian environments. Harsanyi and Selten [14] first explored the question of how to define reasonable bargaining solutions in games with incomplete information. Other attempts were made in the seminal works by Myerson [2, 15, 16], in Maskin and Tirole [17, 18], as well as in Mylovanov and Tröger [19, 20]. Myerson [15] proposed solutions for the problem of mechanism selection by an informed principal who has all of the negotiating ability. This problem was further analyzed as a noncooperative game by Maskin and Tirole [17, 18] for the cases of private and common values. Balkenborg and Makris [10] made connections between Myerson [15] and Maskin and Tirole [18]. For more recent works, see Clark [21] and Peski [22]. Several other authors have addressed the issue of information leakage in mechanism selection games and/or the robustness or stability of mechanisms (see, e.g., [1, 23–29] among many others).

The aforementioned papers share the assumption that the individuals already have their private information when the game begins. This paper considers a richer framework that allows the state of individuals’ information at the mechanism selection stage to be different from that at the implementation stage. The main contribution of this paper is the result that the set of ex ante incentive efficient mechanisms is not robust to changes in the information structure at the mechanism selection stage. This exercise delivers the conclusion that ex ante solutions are sensitive to the specification of what information individuals possess at the time of bargaining, whereas the concept of neutral mechanism is robust to a perturbation of information specification as well as to a possibility of information leakage. This paper solidifies the justification for applying such concept to many bargaining situations with incomplete information. The applications may encompass pretrial negotiations, labor and employment disputes, selling or hiring situations, international conflicts, and bargaining in over-the-counter markets (see, e.g., Kim [12, 13]).

Finally, this paper contributes to the conflict literature on institutional design [30–33]. The literature has addressed several critical questions about the effectiveness of institutions in preventing conflicts. When comparing the performance of different institutions, it may seem natural to focus on institutions that minimize the ex ante likelihood of conflict. Invoking such measure is valid for situations where a conflict-minimizing institution is the only one that would naturally arise, or such an institution is imposed exogenously. But if disputing parties themselves are able to choose among many institutions, a conflict-minimizing institution might not be chosen. The selection of an institution would depend crucially on the information structure at the time of selection. Hence, this paper extends the study of conflict and institutional design by suggesting that the informational environment faced by disputing parties at the time they select an institution should inform which performance measure to use for evaluating institutions.

The remainder of the paper is organized as follows. The next section outlines the model of public goods problems, summarizes the definition of ex ante and interim incentive efficiency, and provides the characterization of neutral mechanisms. The subsequent section presents the main results on ex ante and interim incentive efficient mechanisms, and discusses the advantage of using neutral mechanisms as well as offering the implications for the analysis of ex ante and interim mechanism selections. The last section gives concluding remarks.

## The model

### Setup

The public goods problem is formulated as a Bayesian bargaining problem à la Myerson [2]. Two players, indexed by *i* ∈ {1, 2}, must decide whether or not to produce a discrete public good and how to divide the production cost that is equal to *K* > 0. The set of feasible decisions is $D=\{\left(q,y_{1},y_{2}\right)|0\leq q\leq 1,y_{i}\in R\;\forall i,y_{1}+y_{2}=K\}$, where, for each $(q,y_{1},y_{2})\in D$, *q* represents the probability that the public good is produced and *y*_*i*_ represents player *i*’s share of the cost. Let *y* = (*y*_1_, *y*_2_) denote the profile of cost shares.

For each player *i*, *T*_*i*_ is the finite set of possible types. Each type *t*_*i*_ in *T*_*i*_ represents player *i*’s valuation for the public good. Each player has private information about his type, and has prior beliefs about the other player’s type. For simplicity, I assume that the players’ types are independent random variables. Player −*i* believes that the probability of player *i* being of type *t*_*i*_ ∈ *T*_*i*_ is *p*_*i*_(*t*_*i*_) such that $\sum_{t_{i}\in T_{i}}p_{i}\left(t_{i}\right)=1$. As a regularity condition, I assume that all types have positive probability, so *p*_*i*_(*t*_*i*_) > 0 for all *i* and all *t*_*i*_. Let *T* = *T*_1_ × *T*_2_ denote the set of all possible type combinations *t* = (*t*_1_, *t*_2_). Then the probability that *t* ∈ *T* is the true combination of types for the players is *p*(*t*) ≡ ∏_*i*_ *p*_*i*_(*t*_*i*_).

I assume that the players’ preferences are quasi-linear in (*q*, *y*). Let *u*_*i*_ denote player *i*’s utility function from $D\times T_{i}$ into R, such that *u*_*i*_((*q*, *y*), *t*_*i*_) is the payoff to player *i* of type *t*_*i*_ when (q,y)∈D is chosen. The utility functions are defined by the formula

$$u_{i}(\left(q,y\right),t_{i})=t_{i}q-y_{i},\;\forall i,\;\forall t_{i}.$$

Let $d^{*}\equiv \left(q,y\right)=\left(0,0\right)\in D$ represent the decision not to produce the public good, which is the natural conflict outcome for this problem because no production occurs if the players cannot agree on the division of the cost.

### Feasible and efficient mechanisms

By the revelation principle, I can set up the public goods problem as a direct-revelation mechanism, without loss of generality. That is, the players do not have to agree on a specific decision; instead they may agree on a mechanism.

Because of the linearity of the utility functions, I can restrict attention to deterministic mechanisms, mapping from *T* to D, without loss of generality. So let (*Q*(⋅), *Y*(⋅)) = (*Q*(⋅), *Y*_1_(⋅), *Y*_2_(⋅)) be a *mechanism* for determining the decision as a function of the players’ reported types, where *Q*(*t*) is the probability that the public good is produced and each *Y*_*i*_(*t*) is the expected share of the production cost to be made by player *i* if *t* is the profile of reported types. This mechanism must satisfy 0 ≤ *Q*(*t*) ≤ 1 and *Y*_1_(*t*) + *Y*_2_(*t*) = *Q*(*t*)*K* for all *t* ∈ *T*. If *Q*(*t*) > 0, then *Y*_*i*_(*t*)/*Q*(*t*) represents player *i*’s expected payment per unit of the public good produced when the profile of reported types is *t*. If *Q*(*t*) = 0, any cost payment need not be specified because the public good would not be produced.

Given a mechanism (*Q*, *Y*), let *U*_*i*_(*Q*, *Y*|*t*_*i*_) denote the interim expected utility to type *t*_*i*_ of player *i* given that both players report their types honestly if (*Q*, *Y*) is implemented. That is, for any *i* and any *t*_*i*_ ∈ *T*_*i*_,

$$U_{i}\left(Q,Y|t_{i}\right)=t_{i}\sum_{t_{-i}\in T_{-i}}p_{-i}\left(t_{-i}\right)Q\left(t_{-i},t_{i}\right)-\sum_{t_{-i}\in T_{-i}}p_{-i}\left(t_{-i}\right)Y_{i}\left(t_{-i},t_{i}\right).$$

The implementation of a mechanism is restricted by two constraints of incentive compatibility and individual rationality. If player *i*’s type is *t*_*i*_ but he reports some other type *s*_*i*_ in implementing (*Q*, *Y*), while the other player remains honest, then the expected utility to type *t*_*i*_ of player *i*, denoted by $U_{i}^{*}\left(Q,Y,s_{i}|t_{i}\right)$, is

$$U_{i}^{*}\left(Q,Y,s_{i}|t_{i}\right)=t_{i}\sum_{t_{-i}\in T_{-i}}p_{-i}\left(t_{-i}\right)Q\left(t_{-i},s_{i}\right)-\sum_{t_{-i}\in T_{-i}}p_{-i}\left(t_{-i}\right)Y_{i}\left(t_{-i},s_{i}\right).$$

A mechanism (*Q*, *Y*) is interim *incentive compatible* if and only if $U_{i}\left(Q,Y|t_{i}\right)\geq U_{i}^{*}\left(Q,Y,s_{i}|t_{i}\right)$ for all *i*, for all *t*_*i*_ ∈ *T*_*i*_, and for all *s*_*i*_ ∈ *T*_*i*_.

The conflict outcome d* will occur if the players disagree, and each player has the right to refuse public good production. So no type of any player should expect to do worse under the mechanism, given that both players report their types honestly, than in the conflict outcome. So a mechanism (*Q*, *Y*) is interim *individually rational* if and only if *U*_*i*_(*Q*, *Y*|*t*_*i*_) ≥ 0 for all *i* and for all *t*_*i*_ ∈ *T*_*i*_. Then a mechanism is defined to be *feasible* for the players in this public goods problem if and only if it is both incentive compatible and individually rational. By the revelation principle, there is no loss of generality in focusing on feasible mechanisms.

Given the set of feasible mechanisms, the concept of Pareto efficiency can be applied to identify the entire set of efficient mechanisms among which the players would reasonably choose from. If the mechanism is selected by the players with asymmetric information, then the proper concept of Pareto efficiency is interim incentive efficiency. A mechanism (*Q*, *Y*) is *interim incentive efficient* if and only if (*Q*, *Y*) is feasible and there does not exist another feasible mechanism (*Q*′, *Y*′) such that all types of all players would prefer (*Q*′, *Y*′) over (*Q*, *Y*), that is, *U*_*i*_(*Q*′, *Y*′|*t*_*i*_) ≥ *U*_*i*_(*Q*, *Y*|*t*_*i*_), for all *i* and for all *t*_*i*_ with at least one strict inequality. If the mechanism can be selected before the players learn their private information, then the concept of ex ante incentive efficiency should be applied. A mechanism (*Q*, *Y*) is *ex ante incentive efficient* if and only if (*Q*, *Y*) is feasible and there does not exist another feasible mechanism (*Q*′, *Y*′) such that all players would prefer (*Q*′, *Y*′) over (*Q*, *Y*) before learning their private types, that is, $\sum_{t_{i}\in T_{i}}p_{i}\left(t_{i}\right)U_{i}\left(Q^{\prime },Y^{\prime }|t_{i}\right)\geq \sum_{t_{i}\in T_{i}}p_{i}\left(t_{i}\right)U_{i}\left(Q,Y|t_{i}\right)$, for all *i*, with at least one strict inequality.

### Neutral mechanisms: Characterization

Myerson [2] developed a generalization of the Nash bargaining solution for Bayesian bargaining problems, called the *neutral bargaining solution*. This solution concept is axiomatically defined: A neutral bargaining solution is any mechanism such that it is contained in every solution correspondence that satisfies the probability invariance, extension, and random-dictatorship axioms. Scrutinizing all of the axioms is not the primary goal of the present paper; so without loss of comprehension of the solution concept, I focus only on the random dictatorship axiom. Roughly stated, the probability invariance axiom ensures that the solution is robust to a change in the parameters of the model that does not affect its decision-theoretic structure, and the extension axiom connects solutions to related bargaining problems (see Myerson [2] for more details). The random dictatorship axiom defines a fair and efficient mechanism. This axiom provides the key logic in understanding the neutral bargaining solution, which is stated below in the context of public good problems. Henceforth, I will refer to the neutral bargaining solution as a neutral mechanism or neutral public good mechanism.

**Axiom 1 (Random dictatorship)** *If there exist two interim incentive efficient mechanisms* (*Q*^1^, *Y*^1^) *and* (*Q*^2^, *Y*^2^) *such that U*_2_(*Q*^1^, *Y*^1^|*t*_2_) = 0, *for all t*_2_ ∈ *T*_2_, *and U*_1_(*Q*^2^, *Y*^2^|*t*_1_) = 0, *for all t*_1_ ∈ *T*_1_; *and if the mechanism* (*Q*^*n*^, *Y*^*n*^) *defined by Q*^*n*^(*t*) = 0.5*Q*^1^(*t*) + 0.5*Q*^2^(*t*) *and* *Y*^*n*^(*t*) = 0.5*Y*^1^(*t*) + 0.5*Y*^2^(*t*), *for all t* ∈ *T*, *is interim incentive efficient, then* (*Q*^*n*^, *Y*^*n*^) *is a neutral public good mechanism*.

The hypotheses of Axiom 1 are satisfied for public good problems in which there is a clear mechanism (or public good decision) that each player should demand if he could have all of the bargaining power. In the terminology of Myerson [2], (*Q*^1^, *Y*^2^) and (*Q*^2^, *Y*^2^) in Axiom 1 are called strongly optimal decisions for players 1 and 2 respectively; that is, each of the two “best” mechanisms is the most reasonable solution for the respective player if he could dictatorially choose the mechanism. Then the 50-50 randomization between the two best mechanisms is certainly equitable, and if it is interim incentive efficient, then it is a neutral mechanism.

When the hypotheses of Axiom 1 are satisfied for a given public good problem, or even when the hypotheses are not satisfied but it is clear what the dictatorial mechanism for each player is, then the neutral mechanism is easy to compute. I present a simple class of public good problems which is useful to illustrate an easy characterization of the neutral mechanism.

**Example 1**. To simplify the problem, I restrict my attention to the simple class where $T_{1}=\left\{v_{1}^{h},v_{1}^{l}\right\}$ such that $v_{1}^{h}>v_{1}^{l}$, and *T*_2_ = {*v*_2_}. That is, player 2’s value of the public good is commonly known to be *v*_2_, whereas player 1’s value of the public good depends on his private type, which is unknown to player 2. (For exposition, I use male pronouns for player 1 and female pronouns for player 2.) I call $v_{1}^{h}$ the high type and $v_{1}^{l}$ the low type. I assume that the public good is worth more than it costs regardless of player 1’s type: $v_{1}^{h}+v_{2}>K$ and $v_{1}^{l}+v_{2}>K$. To make the problem interesting, I assume that $v_{1}^{l}<K/2<v_{1}^{h}<K$ and *K*/2 < *v*_2_ < *K*. Let *p*(*h*) and *p*(*l*) denote the prior probabilities of a high type and a low type, respectively, where *p*(⋅) ∈ (0, 1) and *p*(*h*) + *p*(*l*) = 1. Note that because player 2 does not have private information, the *t*_2_ variable in *t* = (*t*_1_, *t*_2_) can be ignored throughout the analysis.

Consider the following feasible mechanism, denoted by (*Q*^1^, *Y*^1^):

$$Q^{1}\left(v_{1}^{h}\right)=Q^{1}\left(v_{1}^{l}\right)=1,\;Y_{1}^{1}\left(v_{1}^{h}\right)=Y_{1}^{1}\left(v_{1}^{l}\right)=K-v_{2},\;Y_{2}^{1}\left(v_{1}^{h}\right)=Y_{2}^{1}\left(v_{1}^{l}\right)=v_{2},$$

in which the players’ expected utilities are

$$U_{1}\left(Q^{1},Y^{1}|v_{1}^{h}\right)=v_{1}^{h}+v_{2}-K,\;U_{1}\left(Q^{1},Y^{1}|v_{1}^{l}\right)=v_{1}^{l}+v_{2}-K,\;U_{2}\left(Q^{1},Y^{1}|v_{2}\right)=0.$$

Under (*Q*^1^, *Y*^1^), the public good is always produced with player 2 paying her whole value *v*_2_, no matter what player 1’s type is, and player 1 paying the remainder to cover the full cost *K*. This mechanism is clearly the best feasible mechanism for both types of player 1, which gives player 2 the expected utility of zero, regardless of the value of *p*(*h*), and so it is interim incentive efficient for any *p*(*h*); therefore (*Q*^1^, *Y*^1^) is the strongly optimal decision for player 1.

Now consider the following two feasible mechanisms, denoted by (*Q*^2^, *Y*^2^) and (*Q*^3^, *Y*^3^):

$$\begin{array}{l} Q^{2}(v_{1}^{h})=1,\;Y_{1}^{2}(v_{1}^{h})=v_{1}^{h},\;Y_{2}^{2}(v_{1}^{h})=K-v_{1}^{h},\;Q^{2}(v_{1}^{l})=0,\;Y_{1}^{2}(v_{1}^{l})=Y_{2}^{2}(v_{1}^{l})=0;\\ Q^{3}(v_{1}^{h})=Q^{3}(v_{1}^{l})=1,\;Y_{1}^{3}(v_{1}^{h})=Y_{1}^{3}(v_{1}^{l})=v_{1}^{l},\;Y_{2}^{3}(v_{1}^{h})=Y_{2}^{3}(v_{1}^{l})=K-v_{1}^{l}, \end{array}$$

in which the players’ expected utilities are, respectively,

$$\begin{array}{l} U_{1}(Q^{2},Y^{2}|v_{1}^{h})=0,\;U_{1}(Q^{2},Y^{2}|v_{1}^{l})=0,\;U_{2}(Q^{2},Y^{2}|v_{2})=p(h)(v_{2}+v_{1}^{h}-K);\\ U_{1}(Q^{3},Y^{3}|v_{1}^{h})=v_{1}^{h}-v_{1}^{l},\;U_{1}(Q^{3},Y^{3}|v_{1}^{l})=0,\;U_{2}(Q^{3},Y^{3}|v_{2})=v_{2}+v_{1}^{l}-K. \end{array}$$

Under (*Q*^2^, *Y*^2^), the public good is produced if player 1 is the high type with player 1 paying his whole value $v_{1}^{h}$ and player 2 paying the remainder, and the public good is not produced if player 1 is the low type. Under (*Q*^3^, *Y*^3^), the public good is always produced with player 1 paying the low type’s value $v_{1}^{l}$ and player 2 paying the remainder, no matter what player 1’s type is.

The characterization of a neutral mechanism depends on the parameter values of the problem. By using Theorem 10.1 in Myerson [34], the entire set of interim incentive efficient mechanisms can be characterized depending on the parameters.

- The case of $p\left(h\right)\left(v_{2}+v_{1}^{h}-K\right)>v_{2}+v_{1}^{l}-K$: Mechanism (*Q*^2^, *Y*^2^) is the best feasible mechanism for player 2 and is an interim incentive efficient mechanism that gives both types of player 1 the expected utility of zero. Therefore, (*Q*^2^, *Y*^2^) is the strongly optimal decision for player 2. Let (*Q*^*n*^, *Y*^*n*^) be the 50-50 randomization between (*Q*^1^, *Y*^1^) and (*Q*^2^, *Y*^2^), that is,
  $$\begin{array}{l} Q^{n}(v_{1}^{h})=1,\;Y_{1}^{n}(v_{1}^{h})=\frac{K-v_{2}+v_{1}^{h}}{2},\;Y_{2}^{n}(v_{1}^{h})=\frac{v_{2}+K-v_{1}^{h}}{2},\\ Q^{n}(v_{1}^{l})=\frac{1}{2},\;Y_{1}^{n}(v_{1}^{l})=\frac{K-v_{2}}{2},\;Y_{2}^{n}(v_{1}^{l})=\frac{v_{2}}{2}, \end{array}$$ (1)
  which is also interim incentive efficient. Then Axiom 1 applies and (*Q*^*n*^, *Y*^*n*^) is a neutral public good mechanism when $p\left(h\right)\left(v_{2}+v_{1}^{h}-K\right)>v_{2}+v_{1}^{l}-K$. Under (*Q*^*n*^, *Y*^*n*^), if player 1 is the high type, then the public good is always produced with player 1 paying $\frac{K-v_{2}+v_{1}^{h}}{2}$ and player 2 paying $\frac{v_{2}+K-v_{1}^{h}}{2}$; if player 1 is the low type, then there is a 50 percent probability of not producing the public good and a 50 percent probability of producing the public good with player 2 paying her whole value *v*_2_ and player 1 paying the remainder. If $v_{1}^{h}=v_{2}$, both players contribute equally to the total cost, paying *K*/2 each, for the production when player 1 is the high type under (*Q*^*n*^, *Y*^*n*^). If $v_{1}^{h}>v_{2}$, then $Y_{1}^{n}\left(v_{1}^{h}\right)>Y_{2}^{n}\left(v_{1}^{h}\right)$, and vice versa otherwise. Thus, the cost shares $\left(Y_{1}^{n}\left(v_{1}^{h}\right),Y_{2}^{n}\left(v_{1}^{h}\right)\right)$ under (*Q*^*n*^, *Y*^*n*^) can be interpreted as the players dividing the total cost “equitably” in the sense that each player pays the amount that is commensurate with his or her value.
- The case of $p\left(h\right)\left(v_{2}+v_{1}^{h}-K\right)\leq v_{2}+v_{1}^{l}-K$: Mechanism (*Q*^2^, *Y*^2^) is not interim incentive efficient because it is dominated by (*Q*^3^, *Y*^3^). So Axiom 1 does not apply because a strongly optimal decision for player 2 does not exist. However, in such case, (*Q*^3^, *Y*^3^) is the best feasible mechanism for player 2 among all interim incentive efficient mechanisms. In fact, although (*Q*^3^, *Y*^3^) is not a strongly optimal decision for player 2, it is clearly the mechanism that player 2 should demand if she has all of the bargaining power to choose. Let (*Q*^*m*^, *Y*^*m*^) be the 50-50 randomization between (*Q*^1^, *Y*^1^) and (*Q*^3^, *Y*^3^), that is,
  $$\begin{array}{l} Q^{m}(v_{1}^{h})=1,\;Y_{1}^{m}(v_{1}^{h})=\frac{K-v_{2}+v_{1}^{l}}{2},\;Y_{2}^{m}(v_{1}^{h})=\frac{v_{2}+K-v_{1}^{l}}{2},\\ Q^{m}(v_{1}^{l})=1,\;Y_{1}^{m}(v_{1}^{l})=\frac{K-v_{2}+v_{1}^{l}}{2},\;Y_{2}^{m}(v_{1}^{l})=\frac{v_{2}+K-v_{1}^{l}}{2}, \end{array}$$ (2)
  which is also interim incentive efficient. Therefore, (*Q*^*m*^, *Y*^*m*^) should be a neutral public good mechanism when $p\left(h\right)\left(v_{2}+v_{1}^{h}-K\right)\leq v_{2}+v_{1}^{l}-K$. Under (*Q*^*m*^, *Y*^*m*^), the public good is always produced with player 1 paying $\frac{K-v_{2}+v_{1}^{l}}{2}$ and player 2 paying $\frac{v_{2}+K-v_{1}^{l}}{2}$ independently of player 1’s type.

The results in Example 1 show that for the class of public goods problems in which (i) strongly optimal decisions exist (so that Axiom 1 applies) or (ii) strongly optimal decisions do not exist but there is a clear mechanism that each player can dictatorially choose, a neutral mechanism for public good provision can be easily computed by the specific formulas given in Eqs (1) and (2) that have clear interpretations. But the hypotheses of Axiom 1 may be restrictive for some public good problems. In such cases, one can appeal to the characterization theorem, given in Myerson [2], for computing neutral mechanisms. Myerson’s theorem proved that a neutral mechanism can be characterized as a feasible mechanism that is not only interim incentive efficient in terms of actual utility payoffs but also both efficient and equitable in terms of transferable virtual-utility payoffs; where a virtual-utility payoff is defined by taking into account the shadow price of the incentive constraints. So a virtual utility for each type of player *i* exaggerates the difference from the types that want to pretend to be player *i*’s type. Myerson established that the neutral mechanism maximizes the sum of the players’ transferable virtual-utility payoffs and allocates the total transferable payoff equally among the players in every state of types; and it gives each player a real expected utility that is at least as large as the limit of virtually equitable allocations for each type, where a virtually equitable allocation balances out conflicting goals of different possible types of player *i*.

## Why neutral mechanisms?

In this section, I show that the use of an ex ante or interim incentive efficient mechanism as the “solution” to public goods problems can be problematic. I justify neutral mechanisms as the reasonable solutions that are both predictively and prescriptively appealing from efficiency and equity standpoints for the production of public goods.

### Ex ante efficient mechanisms

In many practical settings, the public good is worth more than it costs no matter what the individuals’ private valuations are. In terms of the model specification, such settings satisfy *t*_1_ + *t*_2_ > *K* for all *t* = (*t*_1_, *t*_2_) ∈ *T*. In other settings, producing the public good may lead to ex ante social welfare improvement. In all of those environments of public goods problems, the mechanism that minimizes the chance of no production appears to be a natural choice that the players can agree on. To keep the problem’s dimensionality in check, attention can be restricted to the symmetric case where *T*_1_ = *T*_2_ and *p*_*i*_(*s*) = *p*_*j*_(*s*) for all *i*, *j*, and *s* ∈ *T*_1_ = *T*_2_. Ledyard and Palfrey [5] also restricted attention to the symmetric case for part of their analysis. The following result applies to the class of symmetric public goods problems in which the public good is always worth more than its cost.

**Proposition 1** *For symmetric public goods problems with* ∑_*i*_ *t*_*i*_ > *K for all t* ∈ *T*, *the feasible mechanism that maximizes the ex ante probability of public good production is equivalent to the ex ante incentive efficient mechanism*.

*Proof*. The ex ante probability of public good production under mechanism (*Q*, *Y*) is ∑_*t*∈*T*_ *p*(*t*)*Q*(*t*) where *p*(*t*) = ∏_*i*_ *p*_*i*_(*t*_*i*_). Player *i*’s ex ante expected utility can be written as ∑_*t*∈*T*_ *p*(*t*)(*t*_*i*_ *Q*(*t*) − *Y*_*i*_(*t*)). An ex ante incentive efficient maximizes a weighted sum of the players’ ex ante expected utilities subject to feasibility constraints. Because of symmetry, equal weights can be used. Therefore, the sum of the players’ ex ante expected utilities is given by ∑_*i*_∑_*t*∈*T*_ *p*(*t*)(*t*_*i*_ *Q*(*t*) − *Y*_*i*_(*t*)), which can be rewritten as ∑_*t*∈*T*_ *p*(*t*)(∑_*i*_ *t*_*i*_ *Q*(*t*) − ∑_*i*_ *Y*_*i*_(*t*)) = ∑_*t*∈*T*_ *p*(*t*)(∑_*i*_ *t*_*i*_ *Q*(*t*) − *Q*(*t*)*K*) = ∑_*t*∈*T*_ *p*(*t*)(∑_*i*_ *t*_*i*_ − *K*)*Q*(*t*), where ∑_*i*_ *t*_*i*_ > *K* for all *t* ∈ *T*. Then the objective function in the optimization problem of maximizing the sum of ex ante expected utilities subject to feasibility constraints differs from that in the optimization problem of maximizing the ex ante probability of public good production over all feasible mechanisms only by a positive linear transformation. Hence, the equivalence of the solutions follows.

For the ex ante incentive efficient mechanism to be a reasonable prediction that the players would use to help them make public goods decisions, the implied assumption should be that the mechanism is selected at the ex ante stage to be implemented at the interim stage, or that the mechanism is chosen and enforced by an external social planner whose goal is to minimize the chance of no production. Alternatively, one might assume that a social planner who chooses the mechanism is simply uninformed (i.e., does not know the individuals’ private types). In such cases, invoking the ex ante incentive efficient mechanism relies on the strong assumption that the players must be able to commit themselves to the chosen mechanism ex ante.

While this assumption may be valid in some practical settings, there exists another conceptual issue in assuming that players retreat behind the veil of ignorance when the selection is made, regardless of who chooses the mechanism. What if the players are no longer truly ignorant at the time of mechanism selection? Would the selection of an ex ante incentive efficient mechanism still be justified? The ex ante incentive efficient mechanism may be sensitive to the assumption that the players are absolutely certain that nobody has any private information.

To investigate this, I consider a perturbation of the ex ante information structure at the mechanism selection stage. At the moment when players meet initially to decide on a mechanism (or when the selection is made by an external planner), each player has already received his private information *t*_*i*_ with some probability, independently of the other player. I say that mechanism selection is at the *almost ex ante* stage. Formally, I assume that at the almost ex ante stage of mechanism selection each player has probability *ε* ∈ (0, 1) of having learned his type, and a complementary probability, 1 − *ε*, of still waiting to learn his type. Then for any *t*_*i*_ ∈ *T*_*i*_, *εp*_*i*_(*t*_*i*_) is the probability that player *i* already knows his type and the type is *t*_*i*_, and (1 − *ε*)*p*_*i*_(*t*_*i*_) is the probability that player *i* does not know his type but is expected to be of type *t*_*i*_, as would be assessed by player −*i*. This paper’s results do not depend on the assumption of type-independent probability of being informed, which is only for simplicity. For example, let *ε*_*i*_(*t*_*i*_) denote the conditional probability that player *i* will be informed of his type if he was of type *t*_*i*_, for each *t*_*i*_ of player *i*. Then *ε*_*i*_(*t*_*i*_)*p*_*i*_(*t*_*i*_) is the probability that player −*i* would assign to the event that player *i* is informed and is type *t*_*i*_, and (1 − *ε*_*i*_(*t*_*i*_))*p*_*i*_(*t*_*i*_) is the probability that player −*i* would assign to the event that player *i* is uninformed but will be type *t*_*i*_. Note that the marginal probabilities of player *i* being informed and uninformed are respectively $\sum_{t_{i}}\varepsilon_{i}\left(t_{i}\right)p_{i}\left(t_{i}\right)$ and $1-\sum_{t_{i}}\varepsilon_{i}\left(t_{i}\right)p_{i}\left(t_{i}\right)$. All of the results would hold under this specification.

Implementation of the selected mechanism takes place at the standard interim stage, when every player has received his private information (but does not know the other’s information). The players cannot pre-commit themselves to report their types honestly and not to force the conflict outcome in implementing the selected mechanism after every player has learned his type. Therefore, players should choose among the set of available mechanisms that are subject to the feasibility constraints, as is assumed in Myerson’s works. I assume that all feasible mechanisms for a given public good problem are available to players at the selection stage.

In this perturbed setting, the public good mechanism that is expected to arise would be, minimally, incentive efficient in the appropriate sense. The proper concept of efficiency must be based on the players’ evaluations of the anticipated effects of feasible mechanisms. How a player should evaluate a mechanism depends crucially on what information, if any, he possesses at the time of mechanism selection. In my setting, each player may or may not have learned his private information at the almost ex ante stage of selection. For a player who has received private information about his type, mechanisms are evaluated according to his interim preferences. For a player who does not possess any private information, mechanisms are evaluated according his ex ante preferences. The efficient choice of a mechanism at the almost ex ante stage must then be characterized based on all levels of possible interim and ex ante expected utilities for players.

**Definition 1**. *A mechanism* (*Q*, *Y*) *is* almost ex ante incentive efficient (AAIE) *if and only if* (*Q*, *Y*) *is feasible and there does not exist another feasible mechanism* (*Q*′, *Y*′) *such that U*_*i*_(*Q*′, *Y*′|*t*_*i*_) ≥ *U*_*i*_(*Q*, *Y*|*t*_*i*_), *for all i and for all t*_*i*_, *and* $\sum_{t_{i}\in T_{i}}p_{i}\left(t_{i}\right)U_{i}\left(Q^{\prime },Y^{\prime }|t_{i}\right)\geq \sum_{t_{i}\in T_{i}}p_{i}\left(t_{i}\right)U_{i}\left(Q,Y|t_{i}\right)$, *for all i, with at least one strict inequality*.

The almost ex ante notion of incentive efficiency in Definition 1 is a version of Pareto efficiency concepts on the set of feasible mechanisms, the taxonomy for which is developed by Holmström and Myerson [1]. They let $\Delta_{I}^{*}$ denote the set of mechanisms that are interim incentive efficient (IIE). I similarly denote the set of AAIE mechanisms by $\Delta_{AA}^{*}$, which delimits the set of mechanisms that the players could reasonably consider at the almost ex ante stage of mechanism selection. The following equivalence result entails a complete characterization of the set of AAIE mechanisms. The equivalence holds true on any set of classically feasible mechanisms, not just on the set of incentive feasible ones.

**Theorem 1**. *The notion of almost ex ante incentive efficiency is equivalent to the notion of interim incentive efficiency*: $\Delta_{AA}^{*}=\Delta_{I}^{*}$.

*Proof*. Relative to the interim notion of incentive efficiency, Definition 1 has an additional inequality to be satisfied for mechanism (*Q*′, *Y*′) to dominate mechanism (*Q*, *Y*) with respect to uninformed player *i*’s expected utility. For any given mechanism, for each *i*, uninformed player *i*’s expected utility is simply a weighted average of his interim utilities of all possible types. So *U*_*i*_(*Q*′, *Y*′|*t*_*i*_) ≥ *U*_*i*_(*Q*, *Y*|*t*_*i*_), ∀*t*_*i*_, ∀*i* implies $\sum_{t_{i}\in T_{i}}p_{i}\left(t_{i}\right)U_{i}\left(Q^{\prime },Y^{\prime }|t_{i}\right)\geq \sum_{t_{i}\in T_{i}}p_{i}\left(t_{i}\right)U_{i}\left(Q,Y|t_{i}\right),\;\forall i$.

This result can be justified simply by an intuitive reasoning without making recourse to technical proofs. At the almost ex ante stage, each player privately knows his type with probability *ε* ∈ (0, 1). An uninformed player knows that he has yet to learn his type, and his opponent would assign probability 1 − *ε* to this event. Whether a player has observed private information about his type or not is also private information for the player. That is, there are effectively |*T*_*i*_| + 1 number of privately known types of player *i* at the time of mechanism selection: the *t*_*i*_ type for all *t*_*i*_ ∈ *T*_*i*_ and the “uninformed” type. But it is common knowledge that at the implementation stage every player will exactly know his type *t*_*i*_. Because any player’s expected utility in implementing a mechanism depends on the players’ true types, player −*i* would assign probability *εp*_*i*_(*t*_*i*_) + (1 − *ε*)*p*_*i*_(*t*_*i*_) = *p*_*i*_(*t*_*i*_) to the event that *t*_*i*_ is the true type of player *i*, regardless of whether player *i* is informed or not at the selection stage. Thus, the almost ex ante stage becomes essentially identical to the interim stage with an “extended” type set where players have the same probabilistic beliefs over *t*_*i*_-types as they would have at the usual interim stage.

Holmström and Myerson [1] showed that ex ante incentive efficiency implies interim incentive efficiency. With $\Delta_{A}^{*}$ denoting the set of ex ante incentive efficient mechanisms, Theorem 1 has an immediate corollary.

**Corollary 1** *Ex ante incentive efficiency implies almost ex ante incentive efficiency*: $\Delta_{A}^{*}\subseteq \Delta_{AA}^{*}$ .

The equivalence result and the corollary hold for any *ε* ∈ (0, 1). The case of *ε* = 0 corresponds to situations where the mechanism selection is made *ex ante*, before any player’s type is specified. In this case, Holmström and Myerson [1] suggested that the efficient choice of a mechanism will be from the set $\Delta_{A}^{*}$. If there were some chance that a player may have learned his type at the time of selection, even if that chance were vanishingly small, the set of incentive efficient mechanisms that are implementable and reasonable for the players to choose would be enlarged.

Although the demonstration of the results and the underlying intuition are quite simple, their economic significance is large. When the public good appears to be worth more than it costs, the ex ante incentive efficiency seems to be a desirable property that a public good mechanism should have from the perspective of a planner that is interested in minimizing the ex ante probability of production never occurring. In fact, an ex ante incentive efficient mechanism is interim incentive efficient; so from the perspective of players who themselves are choosing a mechanism that is to be implemented ex interim, the ex ante incentive efficient mechanism can be considered a reasonable selection. However, it is vulnerable to any perturbation of the ex ante informational structure at the mechanism selection stage. This result destroys the validity of ex ante incentive efficient mechanisms as the only reasonable choices for the players, and makes the interim notion of incentive efficiency the relevant solution concept.

### Interim efficient mechanisms

Then what would be the reasonable mechanisms that one might expect to actually arise for the production of public goods and are insensitive to variations in the information structure? The simplest natural answer to this question is the whole set of interim incentive efficient mechanisms.

When the mechanism selection is made ex interim, the choice of a mechanism can be determined by an incomplete information bargaining solution (e.g., Harsanyi and Selten [14] and Myerson [2, 15]) applied to the set of mechanisms. Crawford [25] showed one specification of the rules for bargaining over mechanisms that makes any IIE mechanism attainable when mechanism selection takes place at the interim stage. So as a minimal requirement, the players should be expected to choose from the set $\Delta_{I}^{*}$. Even if there is some chance that a player may not have learned his type at the time of selection, by Theorem 1, the players would still choose from the set $\Delta_{I}^{*}$. That is, the set of IIE mechanisms is a set of mechanisms, which the players would reasonably consider, that is robust to any perturbation of the interim informational structure.

But one drawback of characterizing IIE mechanisms is that it identifies too large a set of attainable mechanisms in many settings. Hence, the consideration of the set of IIE mechanisms may not give practical or compelling predictions for public goods problems.

More importantly, when focusing on the set of IIE mechanisms, there is one evident informational issue that implicitly arises during the mechanism selection process. The feasible mechanism that is best for each player depends on whether he is informed or not, as well as on his type if he is informed. Therefore, when the players are discussing which mechanism to implement, demanding a particular IIE (or AAIE) mechanism might convey information about the player’s type; even an uninformed player might be incorrectly identified as being of a certain type by his demand. In that case, the proposed mechanism may no longer be incentive compatible, or the players may refuse to participate. Hence, whether a player is informed or not and no matter what an informed player’s type is, each player should maintain an inscrutable facade in the mechanism selection process (see Myerson [15] for the inscrutability principle). To do so, each player must make some sort of equitable compromise between what he really wants and what he might have wanted if his type had been different, due to the conflicting incentives of different types of the player. Even if a player is uninformed of his true type, he must also express an equitable compromise between all of his possible types.

We must then use an appropriate solution concept that captures the idea of this inscrutable intertype compromise, as well as to refine a possibly large set of IIE mechanisms and get a stronger prediction of public good mechanisms that one might expect to reasonably arise as an outcome of the public good problem. Fortunately, Myerson’s neutral bargaining solution (or neutral mechanism) resolves both the multiplicity and informational issues that arise when using the concept of interim incentive efficiency.

### Neutral mechanisms: Implications

The previous results and discussions highlight several advantages of using neutral mechanisms rather than ex ante or interim incentive efficient mechanisms for the provision of the public good.

First, because the concept of neutral mechanism by definition implies interim incentive efficiency, neutral mechanisms are also robust to alternative specifications of the information structure at the time of mechanism selection, as are interim incentive mechanisms; whereas ex ante incentive efficient mechanisms are not robust. Note that the neutral bargaining solution of Myerson [2] is defined for a class of problems where the information structures are the same at the selection stage as at the implementation stage. If the information structure at the selection stage is perturbed such that there is some chance that a player may not have learned his type, player *i* who is possibly uninformed can be treated as a player who is informed of being the “uninformed” type. Such uninformed-type player has probability *p*_*i*_(*t*_*i*_) of being type *t*_*i*_ for each *t*_*i*_ ∈ *T*_*i*_; so this player’s deliberation of equitable intertype compromise subsumes *t*_*i*_-player’s intertype compromise deliberation for every *t*_*i*_ ∈ *T*_*i*_. Therefore, the neutral bargaining solution’s prescription should be the same for the perturbed settings as for the fully interim settings.

Second, unlike interim incentive efficient mechanisms, neutral mechanisms do not carry the issue of information leakage during the process of mechanism selection or bargaining. The player’s demand of a neutral mechanism is accepted as independent of the player’s type. The need for this inscrutability concern is taken care of by balancing out conflicting goals of different types, which is captured by the conditions for characterizing neutral mechanisms [2].

Further, relative to the concept of interim incentive efficiency, the concept of neutral mechanism gives a stronger prediction of which mechanism should reasonably be chosen. While the set of interim incentive efficient mechanisms may be large, the neutral mechanism is essentially unique if Axiom 1 is satisfied. There is no general uniqueness theorem, but the neutral mechanism is shown to be unique for many classes of symmetric problems given in [2, 12, 13, 34, 35].

Lastly, neutral mechanisms have the desirable properties of efficiency and equity that should be satisfied by a fair and reasonable bargaining solution. These properties, which incorporate the inscrutable intertype compromise concern, are demonstrated by the axioms that the neutral bargaining solution should satisfy or by the conditions in the characterization theorem of Myerson [2].

These features deliver important implications for the analysis of mechanism selection problems.

If ex ante mechanism selection is to be applied to real situations, then players must be absolutely certain that no one is informed of any relevant private information at the stage of mechanism selection. The ex ante incentive efficient mechanism is not robust to adding some uncertainty that a player may be informed of his type; and so it will lose its validity as a reasonable prediction when there is some possibility, even a very small one, that players are not truly ex ante with regard to their private information. Moreover, the players often seek the assistance of a mutually agreed-upon mechanism to help reduce the risk of public good production never occurring that arises precisely because of information asymmetries. Thus, it is more plausible to assume that the players already have their private information at the time they make a decision about which mechanism to use. Even if some player may still be waiting to learn his type at the time of selection, the interim incentive efficiency and neutral bargaining solution concepts’ prescriptions remain unchanged.

When mechanism selection takes place at the interim stage, and if the only concern is achieving Pareto efficiency, the players should be expected to reasonably choose among the set of interim incentive efficient mechanisms. If achieving the property of fairness is also part of the concern, a neutral mechanism can be considered as a reasonable selection; such selection may not be an ex ante incentive efficient mechanism that would have been chosen if the players had selected at the ex ante stage.

Another set of implications concerns the ex ante criterion that is used to evaluate the performance of mechanisms. The proper performance or welfare criterion to evaluate the selected mechanism depends on what information players possess at the time of selection. In terms of the ex ante measures, my result asserts that the almost ex ante solution may maximize neither the ex ante probability of provision nor the ex ante expected gains of the players. On one hand, the ex ante measures should be irrelevant when evaluating the performance of the interim (or almost ex ante) choice of mechanism. On the other hand, the result implies that when evaluating the performance of different mechanisms, it is important to distinguish between situations in which players are allowed to choose their mechanism and those in which they are not; also important is to carefully identify the informational environment that players face when they select a mechanism. Otherwise, ex ante efficiency can be seriously misleading as a welfare measure of the chosen mechanism even if uncertainty about whether players are informed or not is vanishingly small. Also, to evaluate the mechanism’s performance in terms of the ex ante probability of provision may understate the usefulness of the chosen mechanism. The selection of an interim incentive efficient or neutral mechanism may not maximize the ex ante probability of provision, yet it is Pareto efficient and will improve upon unmediated communication or no communication.

## Conclusion

There is no generally accepted interim bargaining solution concept in the literature, but many bargaining situations take place under incomplete information such as mechanism selection problems for public goods environments. This paper provides a more solid grounding for the relevance of mechanism selection at the interim stage to actual public good problems and of Myerson’s concept of neutral bargaining solution to the process of mechanism selection in such problems. I regard the application of the neutral bargaining solution to public goods problems and its determination for my model as major contributions of this paper.

The analysis of ex ante mechanism selection crucially depends on players having absolutely no doubt that all players are ignorant of their types at the time of selection. If that doubt exists, the players may play on each other’s doubt. My contribution is the result that the ex ante incentive efficient solution under the assumption of ex ante selection stage is not robust to a perturbation of the information structure at the selection stage. Further, the set of interim incentive efficient mechanisms can be large, generating infinite predictions of public good mechanisms; and any choice from that set is vulnerable to the possibility of information leakage. I provide the characterization of neutral mechanisms in a class of problems, which yields the tractable set of conditions with interpretations that are insightful, essentially admitting a unique prediction of which mechanism would reasonably arise. These results justify that neutral mechanisms are theoretically appealing and easy to use from a practical perspective in applications to public goods provision.

## References

[1] Holmström B, Myerson RB. Efficient and durable decision rules with incomplete information. Econometrica. 1983;51(6):1799–1819. doi: 10.2307/1912117

[2] Myerson RB. Two-person bargaining problems with incomplete information. Econometrica. 1984;52(2):461–488. doi: 10.2307/1911499

[3] Ledyard JO, Palfrey TR. Voting and lottery drafts as efficient public goods mechanisms. Review of Economic Studies. 1994;61:327–355. doi: 10.2307/2297984

[4] Ledyard JO, Palfrey TR. A characterization of interim efficiency with public goods. Econometrica. 1999;67(2):435–448. doi: 10.1111/1468-0262.00028

[5] Ledyard JO, Palfrey TR. The approximation of efficient public good mechanisms by simple voting schemes. Journal of Public Economics. 2002;83:153–171. doi: 10.1016/S0047-2727(00)00161-4

[6] Ledyard JO, Palfrey TR. A general characterization of interim efficient mechanisms for independent linear environments. Journal of Economic Theory. 2007;133(1):441–466. doi: 10.1016/j.jet.2005.12.006

[7] Gresik TA. Incentive-efficient equilibria of two-party sealed-bid bargaining games. Journal of Economic Theory. 1996;68:26–48. doi: 10.1006/jeth.1996.0002

[8] Wilson R. Incentive efficiency of double auctions. Econometrica. 1985;53(5):1101–1115. doi: 10.2307/1911013

[9] Balkenborg D. Judgment proofness and extended liability in the presence of adverse selection. In: Boyer M, Hiriart Y, Martimort D, editors. Frontiers in the economics of environmental regulation and liability. vol. 91. Ashgate; 2006. p. 265–290.

[10] Balkenborg D, Makris M. An undominated mechanism for a class of informed principal problems with common values. Journal of Economic Theory. 2015;157:918–958. doi: 10.1016/j.jet.2015.02.007

[11] De Clippel G, Minelli E. Two-person bargaining with verifiable information. Journal of Mathematical Economics. 2004;40:799–813. doi: 10.1016/j.jmateco.2003.07.001

[12] Kim JY. Interim third-party selection in bargaining. Games and Economic Behavior. 2017;102:645–665. doi: 10.1016/j.geb.2017.02.013

[13] Kim JY. Neutral bargaining in financial over-the-counter markets. AEA Papers and Proceedings. 2019;109:539–544. doi: 10.1257/pandp.20191100

[14] Harsanyi JC, Selten R. A generalized Nash solution for two-person bargaining games with incomplete information. Management Science. 1972;18(5):80–106. doi: 10.1287/mnsc.18.5.80

[15] Myerson RB. Mechanism design by an informed principal. Econometrica. 1983;51(6):1767–1797. doi: 10.2307/1912116

[16] Myerson RB. Cooperative games with incomplete information. International Journal of Game Theory. 1984;13(2):69–96. doi: 10.1007/BF01769817

[17] Maskin E, Tirole J. The principal-agent relationship with an informed principal: the case of private values. Econometrica. 1990;58(2):379–409. doi: 10.2307/2938208

[18] Maskin E, Tirole J. The principal-agent relationship with an informed principal, II: common values. Econometrica. 1992;60(1):1–42. doi: 10.2307/2951674

[19] Mylovanov T, Tröger T. Informed-principal problems in environments with generalized private values. Theoretical Economics. 2012;7:465–488. doi: 10.3982/TE787

[20] Mylovanov T, Tröger T. Mechanism design by an informed principal: private values with transferable utility. The Review of Economic Studies. 2014;81(4):1668–1707. doi: 10.1093/restud/rdu019

[21] Clark D. The informed principal with agent moral hazard. 2021;Unpublished manuscript.

[22] Peski M. Bargaining with mechanisms. American Economic Review. 2022;Forthcoming.

[23] Celik G, Peters M. Equilibrium rejection of a mechanism. Games and Economic Behavior. 2011;73(2):375–387. doi: 10.1016/j.geb.2011.03.009

[24] Cramton PC, Palfrey TR. Ratifiable mechanisms: learning from disagreement. Games and Economic Behavior. 1995;10(2):255–283. doi: 10.1006/game.1995.1032

[25] Crawford VP. Efficient and durable decision rules: a reformulation. Econometrica. 1985;53(4):817–836. doi: 10.2307/1912656

[26] Laffont JJ, Martimort D. Mechanism design with collusion and correlation. Econometrica. 2000;68(2):309–342. doi: 10.1111/1468-0262.00111

[27] Lagunoff RD. Resilient allocation rules for bilateral trade. Journal of Economic Theory. 1995;66(2):463–487. doi: 10.1006/jeth.1995.1049

[28] Liu Q, Mailath GJ, Postlewaite A, Samuelson L. Stable matching with incomplete information. Econometrica. 2014;82(2):541–587. doi: 10.3982/ECTA11183

[29] Pomatto L. Stable matching under forward-induction reasoning. 2019;Unpublished manuscript.

[30] Bester H, Wärneryd K. Conflict and the social contract. Scandinavian Journal of Economics. 2006;108(2):231–49. doi: 10.1111/j.1467-9442.2006.00452.x

[31] Hörner J, Morelli M, Squintani F. Mediation and peace. Review of Economic Studies. 2015;82(4):1483–1501. doi: 10.1093/restud/rdv022

[32] Kydd A. Which side are you on? bias, credibility, and mediation. American Journal of Political Science. 2003;47(4):597–611. doi: 10.1111/1540-5907.00042

[33] Meirowitz A, Morelli M, Ramsay KW, Squintani F. Dispute resolution institutions and strategic militarization. Journal of Political Economy. 2019;127(1):378–418. doi: 10.1086/700761

[34] Myerson RB. Game theory: analysis of conflict. Cambridge: Harvard University Press; 1991.

[35] Myerson RB. Analysis of two-person bargaining problems with incomplete information. In: Roth AE, editor. Game-theoretic models of bargaining. Cambridge University Press; 1985. p. 115–147.

